# Classical Paroxysmal Nocturnal Hemoglobinuria Presenting With Severe Anemia and Pigmented Acute Kidney Injury

**DOI:** 10.7759/cureus.28448

**Published:** 2022-08-26

**Authors:** Mohith H N, Christopher J Pinto, Jana Poornima, Ajay K Rajput, Marziyeh Bagheri, Basawantrao Patil, Mohammad Nizamuddin

**Affiliations:** 1 Department of Internal Medicine, Karnataka Institute of Medical Sciences, Hubballi, IND; 2 Department of Family Medicine, Spartan Health Sciences University, Vieux Fort, LCA; 3 Internal Medicine, Bushehr University of Medical Sciences, Bushehr, IRN; 4 Department of Internal Medicine, Gadag Institute of Medical Sciences, Gadag, IND

**Keywords:** severe anemia, massive intravascular hemolysis, steroid treatment, on dialysis, paroxysmal nocturnal hemoglobinuria (pnh), rare case report, hematology, hematology disorders

## Abstract

Paroxysmal nocturnal hemoglobinuria is a rare form of intravascular hemolysis caused by an acquired deficiency of complement regulatory glycoproteins. In our case, a 53-year-old male presented with fatigue, discoloration of urine, and reduced urine output. Preliminary investigations showed severe anemia (3.7 g/dl) and hyperkalemia (7.6 mmol/L) in the setting of acute kidney injury, requiring urgent dialysis. Four units of packed cell volumes were transfused for the correction of anaemia. Following initial stabilization, flow cytometry and a fluorescein-labeled proaerolysin (FLAER) study showed a total deficiency of CD59 in 95.92% of granulocytes and a 97.14% deficiency in monocytes. A bone marrow biopsy showed erythroblast hyperplasia confirming the diagnosis of classical paroxysmal nocturnal hemoglobinuria. The patient was treated with steroids, androgens, and iron supplementation and made a complete recovery with a near-total resolution of his acute kidney injury. This paper aims to review the clinical features and investigations in order to focus on acute kidney injury as an outcome of paroxysmal nocturnal hemoglobinuria.

## Introduction

Paroxysmal nocturnal hemoglobinuria (PNH) is a rare form of acquired intravascular hemolysis arising from a somatic mutation involving a gene on the X-chromosome coding for glycosyl phosphatidylinositol-anchored (GPI) complement regulatory proteins [1]. As a result of this mutation, the complement inhibiting protective proteins - C55 (also known as the decay-accelerating factor; DAF) and CD59 (also known as membrane inhibitor of reactive lysis; MIRL) are absent, due to which, red blood cells are constantly lysed by unmediated uncontrolled complement processes [1,2].

This disease is a rare condition with an incidence of roughly 10-16 per million individuals, with a male sex predilection, although the severity of the disease is more in affected females, due to the feature of lyonization [2,3]. Patients usually present with symptoms of fatigue, discoloration of urine, atypical chest pain, and renal dysfunction [1-4]. Initial clinical investigations may show features of intravascular hemolysis with urine samples showing hemoglobinuria [1-4]. Since PNH is also commonly seen in other bone marrow disorders such as aplastic anemia and myelodysplastic syndromes, confirmation of the condition is made with bone marrow analysis and flow cytometry studies [1,2]. Following identification, treatment is with the help of steroids, androgens, and iron replacement [1]. Eculizumab is a C5 complement inhibitor used in the definitive management of the disease but its relatively high cost discourages its use. Novel agents utilizing RNAi therapy and small protein inhibitors are currently undergoing clinical trials [1,2].

## Case presentation

History and physical examination

An Indian gentleman, aged 53, presented to our emergency department in our government-run hospital in Hubballi, Karnataka, India with complaints of easy fatigability for six months, blackish discoloration of his urine for two months, and reduced urine output for the last 10 days. The patient reported being fatigued throughout the day, with intermittently high-colored urine which reduced on increased intake of fluids. The patient visited a non-medical practitioner at the onset of symptoms and was prescribed unknown unlabelled herbal supplements for fatigue. The patient belonged to an agrarian impoverished household with no history of smoking nor working in an industrial lead-based plant. There was no history of fever, bleeding disorders or previous blood transfusions.

**Figure 1:**
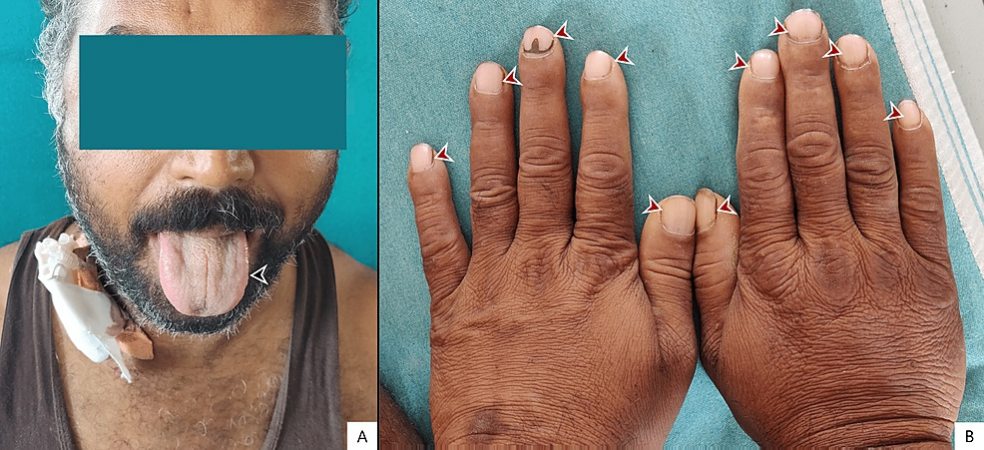
Physical examination findings showing pale tongue and pale nail beds suggestive of anemia. Picture credits: Dr. Mohith H. N

At presentation, the patient had a blood pressure of 102/70 mmHg with a heart rate of 136 bpm, a respiratory rate of 34 cycles per minute, and a saturation of 98%. Physical examination showed a moderately built lethargic male with pale conjunctivae, tongue, and pale nail beds as seen in Figure 1. There was no clubbing, icterus, or cyanosis present. The rest of the physical and systemic examinations were unremarkable.

Emergency department investigations and course

**Figure 2:**
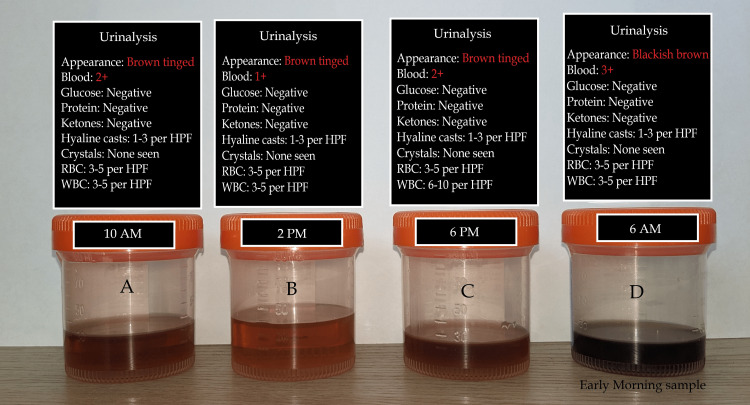
Multiple urine specimens showing visible discoloration demonstrating hemoglobinuria as seen through samples A through D. Picture credits: Dr. Christopher Jude Pinto

Preliminary investigations showed a hemoglobin of 3.7 g/dl, a WBC count of 4620 cells/dl, and a platelet count of 266000 cells/dl. A complete metabolic panel showed hyperkalemia with normal sodium levels (Na+=130 mmol/L, K+=7.6 mmol/L). Renal function tests were abnormal with a serum creatinine level of 15.4 and a BUN of 186. Sequential periodic urinalysis showed hemoglobinuria, few RBCs, and WBCs in all the samples, as seen in Figure 2. Based on the presentation, the diagnosis of severe anemia and acute kidney injury secondary to intravascular processes was made. A differential of drug-induced hemolysis/rhabdomyolysis from the medication prescribed by the non-allopathic quack was also suspected.

In light of the hyperkalemia and suspected drug intravascular hemolysis, the patient was taken in for emergent hemodialysis following administration with calcium gluconate. Following the hemodialysis, the patient was transfused with four units of packed cell volumes abiding by the massive transfusion protocol and was transferred to inpatient care.

Inpatient care investigations and course

**Figure 3:**
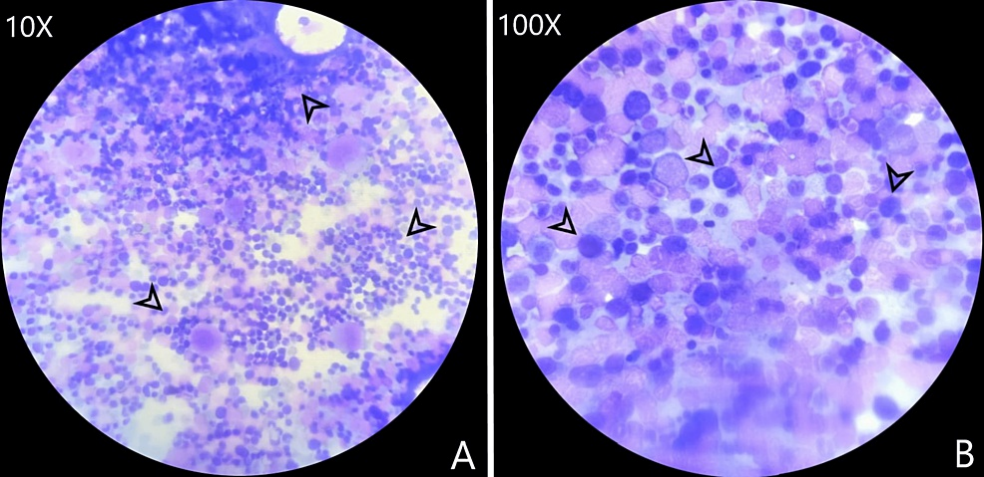
Bone marrow biopsy findings. A. Significant erythroblast proliferation, a few colonies marked in black (10x) (H&E); B. Erythroblasts without any atypia, marked in black (100X) (H&E). Picture credits: Dr. Mohith H. N

Repeat complete blood counts showed persistently declining hemoglobin levels. The intravascular hemolysis panel showed a negative Coombs test with normal complement C3 and C4 levels. Microcytes with a raised reticulocyte count of 7.1% were noted on the peripheral smear. Lactate Dehydrogenase (LDH) levels were significantly raised (3910 IU/L). Haptoglobin levels were abnormally low (2.1). G6PD enzyme levels were normal (20 U/gHb) with the red blood cells showing normal osmotic fragility. Indirect bilirubin levels were raised (2.7 g/dl) with normal direct bilirubin levels (0.8 g/dl). Other liver function tests were within normal limits. The ANA profile was negative. Ultrasound of the abdomen was negative for any abnormalities. Renal ultrasound imaging showed grade 2 parenchymal changes which were suggestive for acute kidney injury. Bone marrow biopsy showed erythroid hyperplasia without any karyotypic abnormalities (Figure 3).

Following extensive investigations of the common causes of intravascular causes of hemolysis, a suspicion for PNH prompted a qualitative assay for CD59 and CD55 via flow cytometry and fluorescein-labeled proaerolysin (FLAER) respectively. Results were significant for 35.5% of RBCs having partial/complete deficiency of CD59, presumably diluted down by the packed cell blood transfusions, however, FLAER showed a total deficiency of CD59 in 95.92% of granulocytes and 97.14% of monocytes (Table 1). The results of the flow cytometry were confirmatory for classical PNH type 3 without the myelodysplastic syndrome, causing severe anemia and pigmented acute kidney injury secondary to hemoglobinuria.

**Table 1:** Flow cytometry and FLAER results showing a near total absence of CD55 within the monocytes and granulocytes and a CD59 deficiency in red blood cells following four units of packed cell transfusions. FLAER: fluorescein-labeled proaerolysin

Flow cytometry	Deficiencies noted	Percentage
Red blood cells (following four units packed cell transfusions)	Partial CD 59 deficiency (Type II cells)	14.80%
	Complete CD59 deficiency (Type III cells)	20.70%
	Total	35.50%
Monocytes (FLAER)	CD55 deficiency	97.14%
Granulocytes (FLAER)	CD55 deficiency	95.92%

The patient was started on daily steroids- prednisolone 30mg, androgens- Danazol 200mg with iron and folic acid supplementation. Eculizumab was not available at our institution. The patient was dialyzed twice weekly for acute kidney injury-induced anuria. A summary of his blood investigations is seen in Table 2.

**Table 2:** Summary of blood investigations done during the emergency visit, inpatient care and post-discharge follow-up. LDH: lactate dehydrogenase, G6PD: Glucose-6-phosphate-dehydrogenase

Blood investigations	ED investigations- Day 1	Post Dialysis and blood transfusions- Day 1	Day 3	Day 7	Day 14	1 month	3 months	6 months
Hemoglobin (g/dl)	3.7	8.1	7.6	8.2	8.4	9.1	10.1	12.1
White Blood Counts (cells/dl)	4620	3900	4200	4400	4000	5000	4700	5200
Platelets (cells/dl)	266000	193000	183000	214000	222000	241000	185000	244000
Blood urea (mg/dl)	186	92	161	60	52	48	38	40
Serum Creatinine (g/dl)	19.1	13.4	11.2	10.7	4.2	2.6	2.2	1.89
Serum Sodium (mmol/L)	130	134	135	138	135	139	140	134
Serum Potassium (mmol/L)	7.6	5.1	6.2	4.2	4.5	3.9	4.1	3.8
LDH (IU/L)	3910	-	-	-	-	-	-	216
Haptoglobin (mg/dl)	2.1	-	-	-	-	-	-	121
G6PD enzyme assay (U/gHb)	20	-	-	-	-	-	-	-
Indirect Bilirubin	2.8	-	-	2.1	2.1	1.9	1.3	0.9
Direct Bilirubin	0.8	-	-	0.8	0.6	0.5	0.6	0.4
Coombs testing	Negative	-	-	-	-	-	-	-

Outcome and follow-up

The patient was discharged on Day 15 of inpatient care following the complete resolution of all symptoms. His subsequent outpatient laboratory investigations showed a marked improved renal function with a normal urine analysis suggestive of resolved hemoglobinuria. Following a treatment period of a month, dialysis was discontinued due to the acute kidney injury being resolved. At his third-month follow-up, his steroids were tapered to an alternate day regimen. At his six-month follow-up, the patient's hemoglobin was 12.1g/dl with a mildly deranged renal function (creatinine - 1.89 mg/dl, blood urea - 40 mg/dl). Currently, the patient is on an alternate-day prednisolone regimen and to date reports to have been symptom-free and is being followed up on every three months.

## Discussion

Paroxysmal nocturnal hemoglobinuria (PNH) has been historically noted in the 1880s from case reports, however, its description was further elucidated by Enneking in 1925 [3]. Later in 1937, Ham demonstrated hemolysis of erythrocytes of individuals with PNH when exposed to an acidified media, hence introducing the test that would be named after him, known as the "Ham test". With the development of modern assays, the Ham test and sucrose lysis were discontinued due to their poor sensitivity and specificity [1,3,5]. Modern assays include the use of flow cytometry and FLAER analysis for detection of CD59 and CD55 respectively. The deficiency of these glycosyl phosphatidylinositol-anchored complement regulatory proteins are responsible for the chronic complement mediated hemolysis seen in this disease process [1-4]. Previously, it was suggested that the disease was "nocturnal" as urine samples during the night and early morning showed intense physical blackish discoloration [6]. One of the theories was that the acidosis during periods of sleep acted as triggers for complement activation and hence an accentuation in colors were noted. This theory was later disproved as hemolysis was shown to occur throughout the day in individuals with PNH, and that the color of the urine was dependent on the hydration status of the individual as seen in our case [6]. Thereby, due to the increased concentration of the urine noted physiologically, overnight and early morning urine samples tend to show a deeper pigmentation visible as brownish-black urine as noted in Figure 2D [6].

As PNH is associated with aplastic anemia and myelodysplastic syndromes, a bone marrow biopsy is necessary for the diagnosis of PNH [1-4,7]. Based on the bone marrow biopsy findings, PNH can be classified into three types can be made as seen in Table 3. An essential test required for confirming the diagnosis of PNH as mentioned previously is the use of flow cytometry and FLAER studies [8-14]. These tests are suggested to be performed with pre-transfusion blood [1,8-14]. In our case, due to the presentation with severely low anemia, blood transfusions were urgently indicated [1]. Following packed cell transfusions, the percentage of RBC deficiency in CD59 could be inaccurate, hence FLAER studies that analyze leukocyte and monocyte CD55 could still be used with high precision [1]. Based on the general findings of the flow cytometry and the FLAER studies, PNH can further be classified, with the more deficient forms having more severe symptom profiles as seen in Table 3 [15]. The minimum required diagnostic tests for confirming PNH are a bone marrow biopsy analysis, cytometric studies (Flow cytometry and FLAER), and supporting laboratory investigations [1]. Supportive laboratory investigations could show a low haptoglobin assay with featuring intravascular hemolysis and a raised LDH level as an outcome of the hemolysis, which was well noted in our case [1].

**Table 3:** Classification of paroxysmal nocturnal hemoglobinuria based on bone marrow biopsy and cytometric findings PNH: paroxysmal nocturnal hemoglobinuria

Classification based on bone marrow biopsy	
Types	Features
Classic PNH	Bone marrow biopsy shows erythroblast hyperplasia without any karyotypic abnormalities
Clinical PNH with aplastic anemia/myelodysplastic syndromes/other unspecified bone marrow disorders	Bone marrow biopsy may show non-random karyotypic and cellular abnormalities. If found, must proceed with chromosome studies. The most common chromosomes involved are 5q, 7q, and 20q.
Subclinical PNH with aplastic anemia/myelodysplastic syndromes/other unspecified bone marrow disorders	Observed in conditions wherein other bone marrow disorders are primary with PNH being a secondary process. As cytometric studies can detect a very small portion of affected cells, an incidental finding may be seen in such a group of diseases.
Classification based on cytometry and FLAER studies	
Types	Features
I	A partial or total deficiency of either CD55 or CD59 was noted in observed cell lineages amounting to <10% of total cells. Commonly seen in subclinical PNH.
II	A partial or total deficiency of either CD55 or CD59 was noted in observed cell lineages amounting to not less than 10% of total cells
III	A partial or total deficiency of either CD55 or CD59 in the majority (>75%) of observed cells

The primary clinical manifestations of PNH are hemolytic anemia (Coombs-negative), cytopenias (in association with aplastic anemia, myelodysplastic syndrome, and other bone marrow disorders), and thrombophilias (in association with intravascular hemolysis and coagulation) [1-4]. These features may not be present in all individuals with PNH due to varying phenotypes among affected individuals. Other clinical features may include acute kidney injury (seen in 14.6% of PNH individuals) [2,4,16], pulmonary hypertension (seen in 1.4% of PNH individuals) [2], dysphagia (seen in 51% of PNH individuals) and erectile dysfunction (seen in 53% of male PNH individuals) [1-4]. Acute kidney injury seen herein could later progress to chronic renal disease (seen in 8.6% of PNH individuals) due to hemosiderin deposition hence, necessitating early diagnosis and treatment [16]. The features of pulmonary hypertension, dysphagia, and erectile dysfunction could be seen due to depleted nitric oxide levels (presumably due to an acquired deficiency caused by the disease process) [1-4]. A thromboembolic phenomenon as a presenting profile in PNH may require anticoagulation to prevent Budd-Chiari syndrome, thrombosis of the renal vein, mesenteric vein, cerebral cavernous veins, and arterial thrombosis [1-4]. However, if the platelet counts and clotting profiles are normal and present without thrombosis, some individuals may not require anticoagulation. The disease is more severe in females, as one X chromosome is inactivated, leaving populations of cells completely deficient in CD55 and CD59 indicating a more active hemolytic process as an outcome of lyonization [1-3].

Acute kidney injury as seen in our case was accompanied by hyperkalemia and raised serum BUN and creatinine. This presentation of acute kidney injury, accompanied by characteristics of hemoglobinuria, required urgent dialysis in the setting of reduced urine output (200ml/day) [4]. In addition to hemoglobin-induced damage to the renal parenchyma, low hemoglobin could have further exacerbated the kidney injury, by reduced oxygen delivery causing compromised renal oxygen perfusion. Following blood transfusions, dialysis, steroids, and androgens herein, the urine output of the patient gradually increased with the gradual normalization of the serum BUN and creatinine with marked improvement in overall kidney function as noted in Table 2.

Definitive treatment options for PNH are limited in application, hence requiring the use of steroids to prevent flares of PNH during episodes of stress, infections, surgery, trauma, and pregnancy [1]. The use of steroids has been noted to help resolve active and chronic hemolytic processes through an unknown mechanism suggesting that steroids herein could be preventive in complement inhibition [17-20]. Though beneficial, the use of steroids is associated with weight gain, myopathies, osteoporotic fractures, and iatrogenic Cushing’s syndrome which may require switching to an alternate-day low-dose regimen [17]. Androgens (Danazol) which was also used in this case, have been noted to aid the hemolytic process by directly stimulating the bone marrow progenitor cells [17,21,22]. Monitoring liver function while the patient is on androgens is imperative for screening for androgen-induced liver hepatotoxicity on a tri-monthly basis [1,17,21,22].

Eculizumab is a monoclonal C5 complement inhibitor, which is used in the definite treatment of PNH, but due to its relatively recent FDA approval in 2007, its availability is limited and its cost prevents its use in South East Asian countries. The use of eculizumab in PNH has shown marked utility by reducing flare-ups, managing acute flare-ups, and improvement in quality-of-life parameters [2,23]. Novel complement inhibitors such as ALXN1210 and RA1011495 are currently under review for the treatment of PNH [2]. Small protein inhibitors such as Coversin and complement D inhibitors such as ACH-4471 are currently in development and are in phase 1 trials [2]. Stem cell transplantation has been debated over its efficacy due to the disease’s heterogenous presentation and its association with bone marrow failure syndromes [1]. Regular follow-up visits with blood investigations for long-term quiescent hemolysis in patients with PNH are warranted, to identify chronic renal disease and thrombophilic events. Patients should be supplemented with iron and folic acid, due to consistent losses noted through quiescent hemolysis and hemoglobinuria [17,22].

## Conclusions

Paroxysmal nocturnal hemoglobinuria is a rare form of acquired intravascular hemolysis arising from a somatic mutation involving a gene in the X-chromosome coding for glycosyl-phosphatidylinositol-anchored complement regulatory proteins. It is a rare condition with an incidence of roughly 10-16 per million individuals. The primary clinical manifestations of PNH are hemolytic anemia, cytopenias, and thrombophilic disorders. Treatment options for PNH are limited in use, hence requiring the use of steroids to prevent flares of PNH during episodes of stress, infections, and pregnancy. Eculizumab, a monoclonal C5 complement inhibitor approved in 2007 by the FDA, is used in the definitive treatment of PNH, but its cost limits its use in South East Asian countries. Regular follow-up visits with blood investigations for long-term quiescent hemolysis in patients with PNH are needed to accurately identify chronic renal disease and thrombophilic events and patients should be supplemented with iron and folic acid due to consistent losses noted through quiescent hemolysis and hemoglobinuria.
